# Prognostic prediction by ^18^F-FDG-PET/CT parameters in patients with neuroblastoma: a systematic review and meta-analysis

**DOI:** 10.3389/fonc.2023.1208531

**Published:** 2023-07-14

**Authors:** Ruimin Hu, Yan Zhang, Siying Liu, Pamela Lee, Chaohong Liu, Aiguo Liu

**Affiliations:** ^1^Tongji Hospital, Tongji Medical College, Huazhong University of Science and Technology, Wuhan, China; ^2^Department of Paediatrics and Adolescent Medicine, Li Ka Shing Faculty of Medicine, The University of Hong Kong, Hong Kong, Hong Kong SAR, China; ^3^Department of Pathogen Biology, School of Basic Medicine, Tongji Medical College, Huazhong University of Science and Technology, Wuhan, China

**Keywords:** neuroblastoma, prognosis prediction, ^18^F-FDG-PET/CT, meta-analysis, SUVmax, MTV, TLG

## Abstract

**Purpose:**

Neuroblastoma is a solid malignant tumor with high malignancy and high risk for metastasis. The prognosis of neuroblastoma ranges from spontaneous regression to insensitivity to therapies and widespread metastasis. There is a non-invasive, panoramic imaging technique called ^18^F-fluorodeoxyglucose (^18^F-FDG) positron emission tomography–computed tomography (PET/CT), which can provide both complete anatomical information *via* CT and extent of FDG uptake value in tumors *via* positron emission detection. PET/CT is a powerful approach to estimating tumoral metabolic activities, and PET/CT parameters have been demonstrated to be associated with the prognosis of various tumors. However, the predictive performance of PET/CT for the prognosis of neuroblastoma remains unclear. This meta-analysis aims to assess the predictive values of maximum standardized uptake value (SUVmax), metabolic tumor volume (MTV), and total lesion glycolysis (TLG) for progression-free survival (PFS), event-free survival (EFS), and overall survival (OS) in neuroblastoma patients.

**Methods:**

Literature in PubMed, Embase, Cochrane Library, and Web of Science from January 1985 to June 2023 was searched for studies evaluating predictive values of PET/CT parameters for the prognosis of neuroblastoma. Search items mainly included “Positron Emission Tomography Computed Tomography” and “Neuroblastoma”. Hazard ratio (HR) was used as a pooled statistic to assess the association of SUVmax, MTV, and TLG with PFS, EFS, and OS in neuroblastoma patients. Heterogeneity test and sensitivity analysis were performed.

**Results:**

There were eight studies included, with 325 participants. Meta-analysis showed that higher SUVmax was associated with shorter OS [HR = 1.27, 95% CI (1.11, 1.45), p = 0.001], while no association with PFS [HR = 1.03, 95% CI (0.99, 1.07), p = 0.222] and EFS [HR = 2.58, 95% CI (0.37, 18.24), p = 0.341] was presented. MTV showed no association with OS [HR = 2.46, 95% CI (0.34, 18.06), p = 0.376] and PFS [HR = 2.60, 95% CI (0.68, 9.88), p = 0.161]. There was a statistically significant association between TLG and OS [HR = 1.00, 95% CI (1.00, 1.00), p = 0.00], while the HR was 1, so the association could not be concluded, and TLG showed no association with PFS [HR = 1.00, 95% CI (0.99, 1.00), p = 0.974].

**Conclusion:**

High SUVmax indicates poor OS in patients with neuroblastoma. The MTV and TLG are potential prognostic predictors that need to be further validated by more well-designed studies.

**Systematic review registration:**

https://www.crd.york.ac.uk/PROSPERO/, identifier 340729.

## Introduction

Neuroblastoma (NB) is a solid malignant tumor that prevalently occurs in the extracranial sympathetic nervous system in children (1). It accounts for approximately 15% of pediatric cancer fatalities due to its high malignancy and high risk for metastasis (2). Despite advances in multi-modal therapies including dose-intensive and myeloablative therapy with hematopoietic stem cell support, radiotherapy, and immunotherapy, the survival of children with metastatic neuroblastoma remains poor (International Neuroblastoma Risk Group Staging System [INRGSS] Stage M), with a 3-year event-free survival of 60% (3). The prognosis of neuroblastoma varies from individual to individual, ranging from spontaneous regression to insensitivity to therapies and widespread metastasis (4). Accurate predictors would be of great significance for risk stratification and individualized management for neuroblastoma patients so as to improve their prognosis.

^18^F-Fluorodeoxyglucose (^18^F-FDG) positron emission tomography/computed tomography (PET/CT) is a non-invasive, panoramic imaging technique that can provide complete anatomical information *via* CT and detect the extent of FDG uptake in primary tumors and metastases (5). Maximum standardized uptake value (SUVmax) is the most commonly used PET/CT parameter for the estimation of tumoral metabolic activities, which has been demonstrated to be associated with the prognosis of various tumors. Several volumetric imaging parameters based on ^18^F-FDG PET/CT, including metabolic tumor volume (MTV) and total lesion glycolysis (TLG), have also been recommended as prognostic factors for various tumors (6–10). For example, TLG with a cutoff value of 443.8 is significantly associated with the overall survival (OS) of patients with small cell lung cancer (6). A study has shown that SUVmax is significantly associated with modified Bloom-Richardson (MBR) grades in patients with triple-negative breast cancer (TNBC) (7). It has been reported that patients with high SUVmax often have poorer survival outcomes (7). A meta-analysis has indicated that SUVmax measured before treatment and its metabolic response after treatment are of predictive value for the long-term survival of head and neck cancer (8). Another two meta-analyses have concluded that high SUVmax, MTV, and TLG indicate a higher risk for recurrence or death in patients with pancreatic carcinoma (9) and patients with surgical non-small cell lung cancer (10). Despite the increasing application of ^18^F-FDG PET/CT in pediatric neuroblastoma for diagnosis, staging, and prognosis prediction (11–14), the consistency of SUVmax and volumetric PET parameters remains elusive in prognosis prediction of neuroblastoma. Therefore, we have conducted this systematic review and meta-analysis to assess the predictive values of ^18^F-FDG PET/CT-based metabolic parameters for survival outcomes in patients with neuroblastoma.

## Materials and methods

This study is conducted in strict accordance with the Preferred Reporting Items for Systematic Reviews and Meta-Analyses (PRISMA) guidelines (15).

### Literature search and study selection

PubMed, Embase, Web of Science, and Cochrane Library were searched from January 1985 to June 2023 for relevant studies, with language restriction to English. Search items mainly contained the following: (“Neuroblastoma” or “Neuroblastomas”) and (“Positron Emission Tomography Computed Tomography”). The detailed search strategy is shown in the **Supplementary Material**.

Studies meeting the following criteria were included: observational study (prospective and retrospective) or clinical trial that applied ^18^F-FDG PET/CT and relevant parameters (SUVmax, MTV, and TLG) in NB patients and reported survival data, such as OS, progression-free survival (PFS), and event-free survival (EFS).

Literature review, conference summary, case report, and editorial materials were excluded.

Literature search and study selection were conducted by two reviewers independently, and disagreements were settled *via* discussion.

### Quality assessment and data extraction

Quality assessment of included studies was performed by two reviewers independently using the Quality in Prognostic Studies (QUIPS) tool (16) *via* Review Manager 5.4 software. QUIPS contains six domains: study participation, study attrition, measurement of prognostic factors, measurement of outcome, study confounding, and statistical analysis and reporting. Disagreements were settled *via* discussion.

Data were extracted independently by two reviewers using a pre-designed form that included the following: name of the first author, publication date, sample size, country, study design, characteristics of participants (gender distribution, tumor grade, tumor site, treatment after PET/CT scans, volumes of interest (VOIs) for recording SUVmax, and reported survival), PET parameters, and cutoff values of parameters.

### Statistical analysis

The primary endpoint was OS, defined as the time interval from the initiation of treatment to all-cause death. The secondary outcome was PFS, referring to recurrence-free survival and the time interval from the date of treatment initiation to tumoral recurrence or metastasis. EFS was calculated from diagnosis to the first occurrence of relapse, progression, secondary malignancy, death, or the last contact if no event occurred. Hazard ratio (HR) was applied as the statistic for the association of SUVmax, MTV, or TLG with PFS, EFS, and OS. PFS, EFS, or OS data were extracted using methods mentioned previously (17). Univariate or multivariate HR with a 95% confidence interval (95% CI) were extracted from each study if provided; otherwise, Engauge Digitizer would be applied (http://markummitchell.github.io/engauge-digitizer/) to estimate the survival rate through Kaplan–Meier curve and reconstruct HR estimate and its variance, assuming that patients were censored at a constant rate during the follow-up. A heterogeneity test was performed using chi-square (χ^2^) test and I^2^statistic (18). I^2^ less than 50% with a p-value not less than 0.1 indicated no significant heterogeneity among the studies, and a fixed-effects model would be applied; otherwise (I^2^ greater than 50% with a p-value less than 0.1), a random-effects model would be applied. Meanwhile, sensitivity analysis was performed by removing each included study one by one to assess the robustness of the results. Statistical analysis was performed using Stata Version 16.0 (College Station, TX, USA). A p-value less than 0.05 indicated statistical significance.

## Results

### Characteristics of included studies

**Figure 1:**
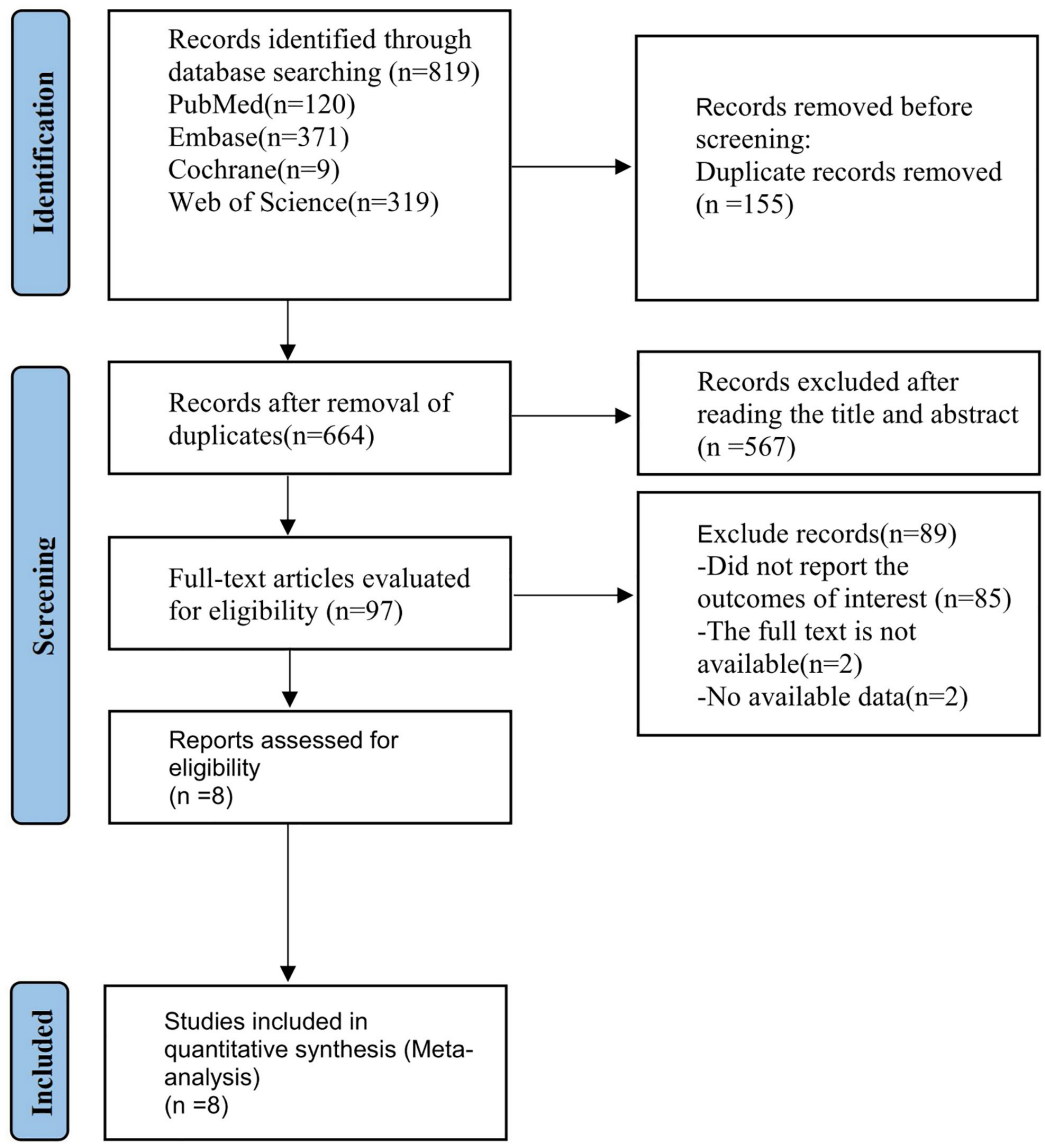
PRISMA flow diagram of the study process. PRISMA, Preferred Reporting Items for Systematic Reviews and Meta-Analyses.

The flow diagram of the study selection process is presented in **Figure 1**. A total of eight studies, involving 325 participants, were included, among which seven studies (13, 19–24) were retrospective design and one study (25) was prospective. According to the INRGSS grade, one study (25) only recruited patients with stage IV neuroblastoma; one study recruited those at stages I, II, and IV (20); four studies recruited patients at all grades (13, 19, 21, 24); the remaining two studies failed to clearly describe the grading of the patients (22, 23). There were six studies that included neuroblastoma originating in the adrenal glands, retroperitoneum, and mediastinum (19–24), and the other two studies (13, 25) failed to clearly state the tumor sites. The characteristics of included studies are shown in **Table 1**. All the studies used ^18^F-FDG for PET imaging, among which seven studies reported SUVmax (13, 19, 21–25), four studies reported MTV and TLG (13, 20, 22, 24), four studies reported the predictive value of SUVmax for OS (19, 21, 23, 25), three studies reported association of SUVmax with PFS (or recurrence-free survival) (13, 19, 22), two studies reported association of SUVmax with EFS (21, 24), two studies reported association of MTV and TLG with OS (20, 22), and three studies reported the predictive value of MTV and TLG for PFS (13, 20, 22). One study (19) provided spheroid-shaped VOI for the primary tumor lesion and metastatic lesions of each patient to evaluate FDG uptake of neuroblastoma lesions, and SUVmax in each VOI was measured, while another study measured SUVmax in VOI for the most intense lesion (25). The cutoff value of SUVmax ranged from 3.31 to 12.01, and those of MTV in two studies (20, 22) were 88.1 and 191 cm^3^, respectively. The cutoff values of TLG in two studies (2, 5) were 1,045.2 and 341.41 g, respectively.

**Table 1:** Characteristics of the included study.

No.	Study	Year	Patient no.	Country	Study design	Male (%)	Stage	Tumor location	Treatment	VOI for recording SUVmax	Studied PET parameters	Endpoint	Cutoff value
1	Nikolaos	2011	28	The United Kingdom	Prospective	57.14	IV	(–)	High-dose ^131^I-MIBG and topotecan	Most intense lesion	^18^F-FDG uptake pattern, SUVmax, ^18^F-FDG skeletal extent score	OS	SUVmax = 5.3
2	Lee	2015	50	South Korea	Retrospective	68	I–IVs	Adrenal gland:retroperitoneum:mediastinum = 36: 9: 5	Chemotherapy, surgical resection, PBSCT, radiotherapy, I-131 MIBG	Primary tumor lesions and metastatic lesions	Pmax, Tmax, Tmax/Lmean	OS, PFS	SUVmax = 4
3	Li	2018	47	China	Retrospective	68.09	I, II, IV	Adrenal:paraspinal:periaortic regions = 16:2:29	(-)	Primary tumor lesion	SUVmax,MTV, TLG, BMU patterns	RFS, OS	SUVmax = 4.15; TLG = 10,454.2 g; MTV = 88.1 cm^3^
4	Liu	2017	25	China	Retrospective	80	I–IV	(-)	(-)	Primary tumor lesion	SUVmax,MTV, TLG	OS, PFS	(-)
5	Liu	2016	42	China	Retrospective	66.67	I–IVs	Adrenal : RP/Med = 29: 11/2	(-)	Primary tumor lesion	SUVmax	OS, EFS	SUVmax = 3.31
6	Man	2021	40	China	Retrospective	47.5	(-)	Retroperitoneal:mediastinal:other = 31:7:2	Comprehensive treatment	Primary tumor lesion	SUVmax, MTV, TLG	OS, PFS	SUVmax = 12.01; TLG = 341.41 g; MTV = 191 cm^3^
7	Sung	2020	55	The USA	Retrospective	52.7	(-)	Adrenal:extra-adrenal = 33: 22	(-)	Primary tumor lesion	SUVmax, SUVmax/SUVliver, SUVmean	OS	SUVmax = 4.77
8	Liu	2022	38	China	Retrospective	36.8	I–IVs	Abdomen:non-abdomen = 34:4	Chemotherapy, surgery	Primary tumor lesion	SUVmax, MTV, TLG	EFS	(-)

SUVmax, maximum standardized uptake value; MTV, metabolic tumor volume; TLG, total lesion glycolysis; PBSCT, autologous peripheral blood stem cell transplantation; VOI, volume of interest; BMU patterns, bone marrow uptake patterns; Pmax, the SUVmax of the primary tumor lesion; Tmax, the SUVmax of all the tumor lesions including the primary tumor lesion and metastatic lesions; Tmax/Lmean, the uptake ratio of Tmax to mean SUV of normal liver tissue; OS, overall survival; PFS, progression-free survival; RFS, recurrence-free survival; EFS, event-free survival.

### Quality assessment

**Figure 2:**
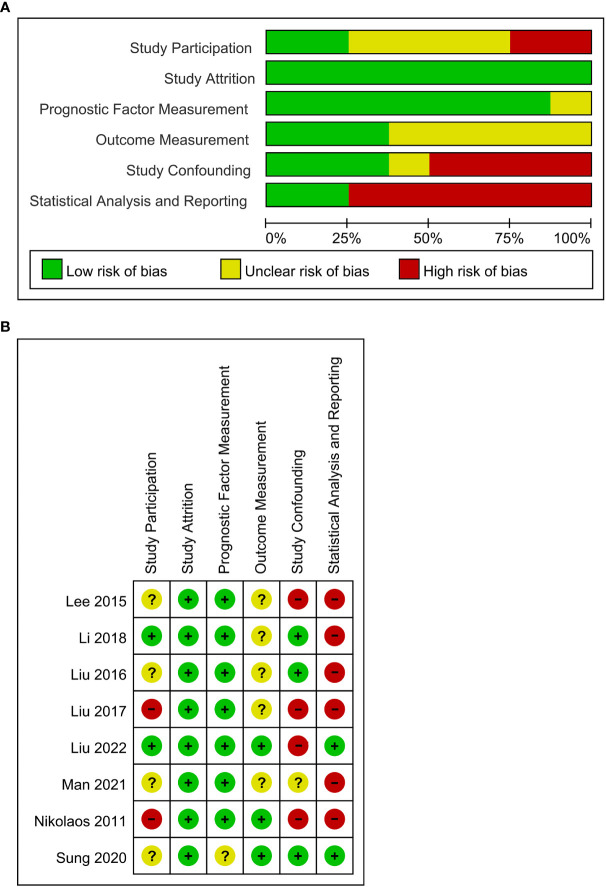
**(A)** QUIPS risk of bias graph: the judgments about each risk of bias domain are presented as percentages across all included studies (n = 8). **(B)** Summary of quality assessment of individual studies according to Quality in Prognostic Studies (QUIPS).

There were four studies (19, 21–23) that were graded as unclear in selection bias due to no description of consecutive selection for participants, one study (13) was graded as high selection bias due to limited sample size, and one study (25) was graded as high selection due to recruitment of only stage IV patients. All the studies were graded as low risk of attrition bias. There was one study graded as unclear risk of bias in prognostic factor measurement (23) because it failed to state the participation of two experienced nuclear medicine physicians in the measurement. There were five studies (13, 19–22) graded as having unclear risk of bias in outcome measurement due to no description of detailed methods for measurement. There were four studies (13, 19, 24, 25) graded as high risk in confounding bias due to the lack of multivariate analysis and one study (22) due to an unclear risk because it performed both multivariate analysis and univariate survival analysis. In terms of statistical analysis and reporting, six studies (13, 19–22, 25) were graded as high risk of bias in that these studies failed to provide the HRs of non-significant factors. The overall quality of included studies was moderate (**Figure 2**).

### Predictive value of SUVmax, MTV, and TLG on PFS, EFS, and OS

**Figure 3:**
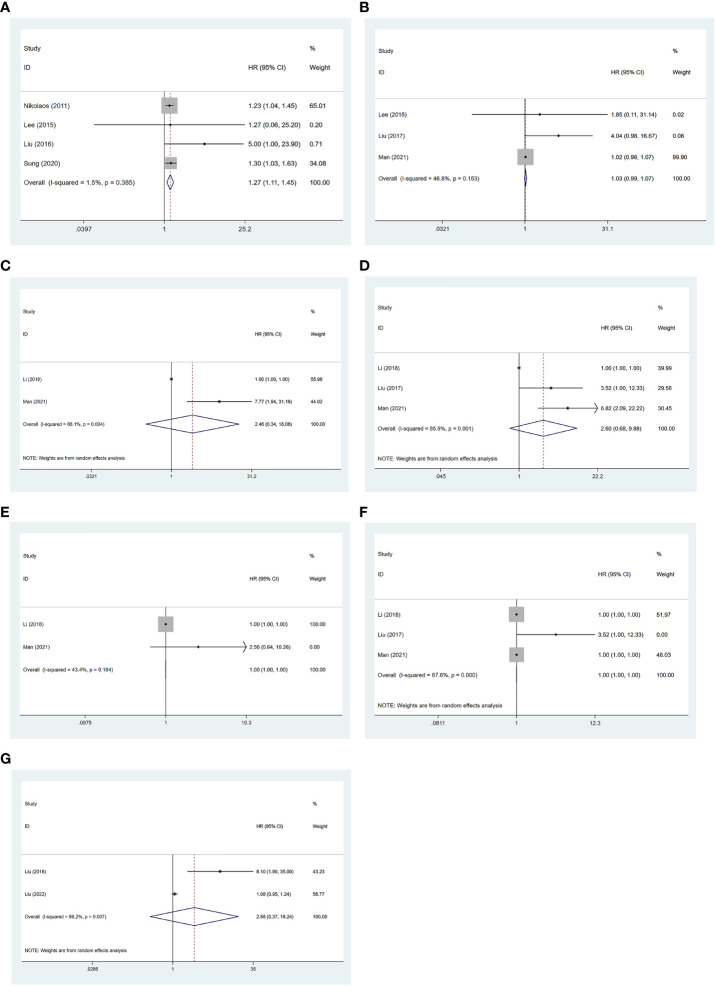
Forest plot results of the OS based on SUVmax **(A)**, MTV **(C)**, and TLG **(E)**; PFS based on SUVmax **(B)**, MTV **(D)**, and TLG **(F)**; and EFS based on SUVmax **(G)**. OS, overall survival; SUVmax, maximum standardized uptake value; MTV, metabolic tumor volume; TLG, total lesion glycolysis; PFS, progression-free survival; EFS, event-free survival.

There were four studies that reported an association of SUVmax with OS (19, 21, 23, 25). No significant heterogeneity was considered among the studies (I^2 = ^1.5%), followed by a fixed-effects model applied. Meta-analysis showed that the value of SUVmax was negatively associated with the OS of NB patients [HR = 1.27, 95% CI (1.11, 1.45), p = 0.001] (**Figure 3A**).

There were three studies that reported the association of SUVmax with PFS (13, 19, 22). No significant heterogeneity was considered among the studies (I^2 = ^46.8%, p = 0.153), followed by the fixed-effects model applied. Meta-analysis showed no significant association between SUVmax and the PFS of NB patients [HR = 1.03, 95% CI (0.99, 1.07), p = 0.222] (**Figure 3B**).

There were two studies that reported the association of MTV with OS (20, 22). Significant heterogeneity was observed (I^2 ^=^ ^88.1%, p = 0.004), so a random-effects model was used. Meta-analysis showed no significant association between MTV and the OS of NB patients [HR = 2.46, 95% CI (0.34, 18.06), p = 0.376] (**Figure 3C**).

There were three studies that reported an association of MTV with PFS (13, 20, 22). Significant heterogeneity was observed (I^2 ^=^ ^85.8%, p = 0.001), and a random-effects model was used. Meta-analysis showed no significant association between MTV and the PFS of NB patients [HR = 2.60, 95% CI (0.68, 9.88), p = 0.161] (**Figure 3D**).

There were two studies that reported the association of TLG with OS (20, 22). No significant heterogeneity was considered among the studies (I^2 ^=^ ^43.4%, p = 0.184), and a fixed-effects model was applied. Meta-analysis showed that TLG was significantly associated with the OS of NB patients [HR = 0.99, 95% CI (0.99, 0.99), p = 0.00] (**Figure 3E**).

There were three studies that reported the association of TLG with PFS (13, 20, 22). Significant heterogeneity was observed (I^2 ^=^ ^87.6%, p = 0.000), and a random-effects model was applied. Meta-analysis showed no significant association between TLG and the PFS of NB patients [HR = 1.00, 95% CI (1.00, 1.00), p = 0.974] (**Figure 3F**).

There were two studies that reported the association of SUVmax with EFS (21, 24). Significant heterogeneity was observed (I^2 ^=^ ^86.2%, p = 0.007), so a random-effects model was used. Meta-analysis showed no significant association between SUVmax and the EFS of NB patients [HR = 2.58, 95% CI (0.37, 18.24), p = 0.341] (**Figure 3G**).

### Sensitivity analysis

A sensitivity analysis was performed (**Supplementary Figure S1**) to assess the robustness of the results. Since the research data on OS based on MTV, EFS based on SUVmax, and OS based on TLG are relatively small, only sensitivity analysis was performed on OS based on SUVmax, PFS based on SUVmax, PFS based on MTV, and PFS based on TLG. Among studies of SUVmax on OS, the combined HRs were found to be stable, suggesting that no individual study significantly affected the results (**Supplementary Figure S1A**). Of all studies of SUVmax on PFS, one study (22) had a great impact on the results. After this study was excluded, the combined HR was far larger than before. As for studies of MTV on PFS, the combined HRs were also found to be stable, indicating that no individual study significantly affected the results. Among studies of TLG on PFS, after excluding one study (13), the value of HR remained unchanged. This indicated that this study had no effect on the results.

## Discussion

In this meta-analysis, we have found that a higher SUVmax value of ^18^F-FDG PET/CT would be associated with a higher mortality risk in patients with neuroblastoma, while its predictive performance for PFS and EFS still needs to be further validated. MTV and TLG present no predictive significance for either the PFS or OS of those patients.

SUVmax is the most commonly used ^18^F-FDG PET/CT parameter for disease diagnosis and treatment response monitoring due to its high repeatability and availability. In our review, four HRs regarding SUVmax on OS were combined. SUVmax has been shown to be of predictive effect despite the thresholds of SUVmax varying among the studies. No significant heterogeneity was observed among the four studies (19, 21, 23, 25), and sensitivity analysis indicated the robustness of the results. However, the predictive effect of SUVmax for PFS and EFS could not be concluded, which might be attributed to insufficient data, limited number of included studies, and varied methods for outcome measurement.

This study has revealed that MTV was not superior to SUVmax regarding the prediction of PFS and OS, which could be explained by several reasons. First, the three included studies (13, 20, 22) regarding MTV had recruited too few patients to produce conclusive results. Then, MTV represents the size of tumor tissues that exhibit active ^18^F-FDG uptake, which makes it unreliable and unrepeatable, especially for multiple, disseminated, and extensive lesions. Moreover, there is a lack of standardized measuring procedures for estimating MTV thresholds. Chao Li et al. (20) and Chia-Ju Liu (13) have estimated MTV thresholds based on 40% of the SUVmax, whereas Shuai Man et al. (22) have used 42% of the SUVmax. Using a proportion of the SUVmax as a threshold may lead to a misestimation of the calculated tumor volume in cases of heterogeneous or low uptake. There is a study reporting that an individualized threshold based on the liver background could reduce the impact of different scanning techniques on solid tumor-associated indicators (26). Thus, a standardized measuring method for MTV is needed for more accurate assessments in patients with neuroblastoma.

TLG is an ideal metabolic parameter that combines the mean SUV value and MTV to assess tumor volume and metabolism. This study has yielded results inconsistent with those of previous studies (9, 27), which have demonstrated the predictive value of TLG for patients with pancreatic carcinoma or extranodal natural killer/T-cell lymphoma. The possible reasons might be related to the limited number of studies included and the TLG calculations and estimation subjecting SUVmax and MTV, which are affected by MTV measurement methods.

This study has several limitations. First, there are insufficient data to properly assess the predictive performance of MTV and TLG for the patients’ prognosis, and some of the studies have only performed univariate analysis leading to potential confounding factors in their results. Second, most of the included studies were retrospective, with moderate methodological qualities. Finally, variances exist in study design, imaging analysis, cutoff value, and inclusion and exclusion criteria for patient recruitment among the included studies, which might lead to heterogeneity.

More well-designed studies with larger samples would be needed for further assessment.

## Conclusion

The SUVmax of ^18^F-FDG PET/CT is of significant predictive effect on the prognosis of neuroblastoma patients. A high SUVmax is associated with a poorer survival prognosis in neuroblastoma patients. In the future, the SUVmax of ^18^F-FDG PET/CT could be used as a predictor for prognosis in patients with neuroblastoma.

## Data availability statement

The original contributions presented in the study are included in the article/**Supplementary Material**. Further inquiries can be directed to the corresponding author.

## Author contributions

All authors contributed to the study’s conception and design. RH and YZ: conceptualization, methodology, software, writing—original draft, data curation, and visualization. SL and PL: investigation, writing—original draft, and writing—reviewing and editing. CL: methodology, software, and writing—original draft. AL: conceptualization, supervision, project administration, and funding acquisition. All authors read and approved the final manuscript.

## Supplementary material

Supplementary Figure S1 Results of sensitivity analysis in OS based on SUVmax **(A)**, PFS based on SUVmax **(B)**, MTV **(C)**, TLG **(D)**.
