# Supplementary material for: Prognostic prediction by 18F-FDG-PET/CT parameters in patients with neuroblastoma: a systematic review and meta-analysis
Source: Front Oncol. 2023 Jul 14;13:1208531. doi: 10.3389/fonc.2023.1208531 (PMC10375790; doi:10.3389/fonc.2023.1208531)
Supplement: Supplementary file 1 [file Table_1.docx]

**Population（P）：** neuroblastoma

**Subject Heading：Neuroblastoma**

**Textwords：****Neuroblastomas**

**Intervention（I）：** PET/CT

**Subject Heading：****Positron Emission Tomography Computed Tomography**

**Textwords**：

PET-CT Scan

PET-CT Scans

Scan, PET-CT

Scans, PET-CT

PET CT Scan

CT Scan, PET

CT Scans, PET

PET CT Scans

Scan, PET CT

Scans, PET CT

CT PET

Positron Emission Tomography-Computed Tomography

PET-CT

CT PET Scan

CT PET Scans

PET Scan, CT

PET Scans, CT

Scan, CT PET

Scans, CT PET

**Pubmed:**

((Positron Emission Tomography Computed Tomography[MeSH Terms]) OR (((((((((((((((((((PET-CT Scan[Title/Abstract]) OR (PET-CT Scans[Title/Abstract])) OR (Scan, PET-CT[Title/Abstract])) OR (Scans, PET-CT[Title/Abstract])) OR (PET CT Scan[Title/Abstract])) OR (CT Scan, PET[Title/Abstract])) OR (CT Scans, PET[Title/Abstract])) OR (PET CT Scans[Title/Abstract])) OR (Scan, PET CT[Title/Abstract])) OR (Scans, PET CT[Title/Abstract])) OR (CT PET[Title/Abstract])) OR (Positron Emission Tomography-Computed Tomography[Title/Abstract])) OR (PET-CT[Title/Abstract])) OR (CT PET Scan[Title/Abstract])) OR (CT PET Scans[Title/Abstract])) OR (PET Scan, CT[Title/Abstract])) OR (PET Scans, CT[Title/Abstract])) OR (Scan, CT PET[Title/Abstract])) OR (Scans, CT PET[Title/Abstract]))) AND ((Neuroblastomas[Title/Abstract]) OR (Neuroblastoma[MeSH Terms]))

**Embase:**


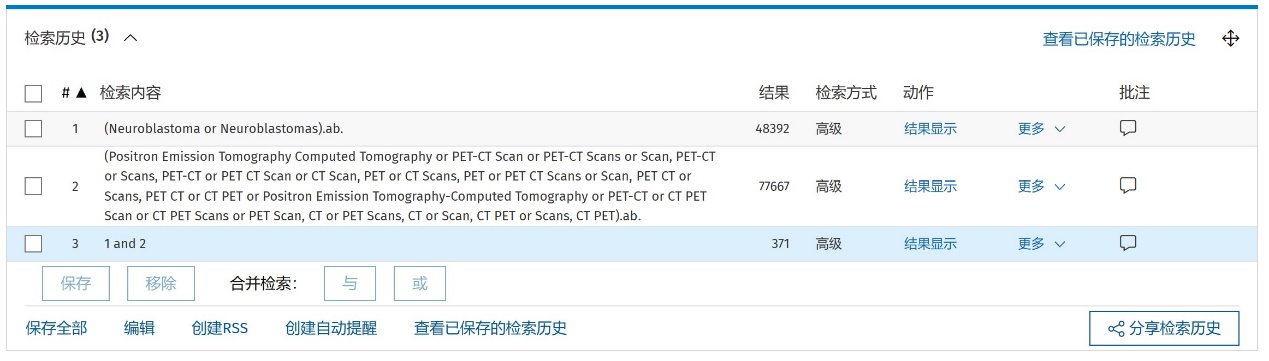


**Web of Science:**


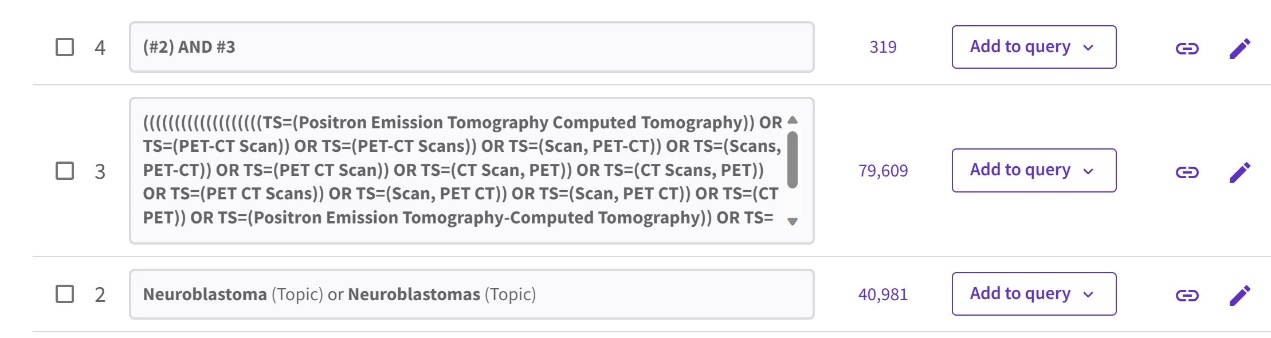


**Cochrane library:**

**
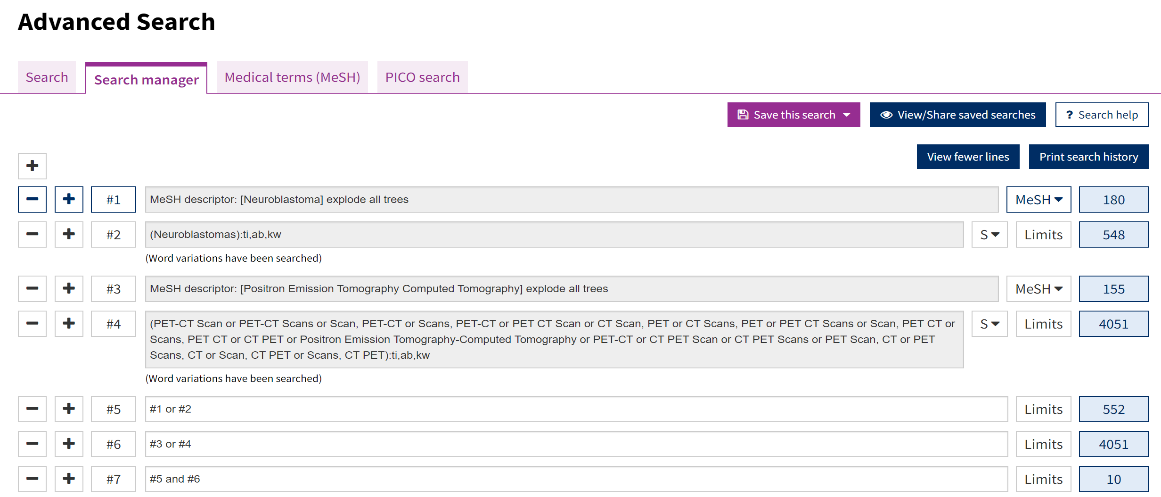
**
