# Supplementary figures and images for: Prognostic prediction by 18F-FDG-PET/CT parameters in patients with neuroblastoma: a systematic review and meta-analysis
Source: Front Oncol. 2023 Jul 14;13:1208531. doi: 10.3389/fonc.2023.1208531 (PMC10375790; doi:10.3389/fonc.2023.1208531)

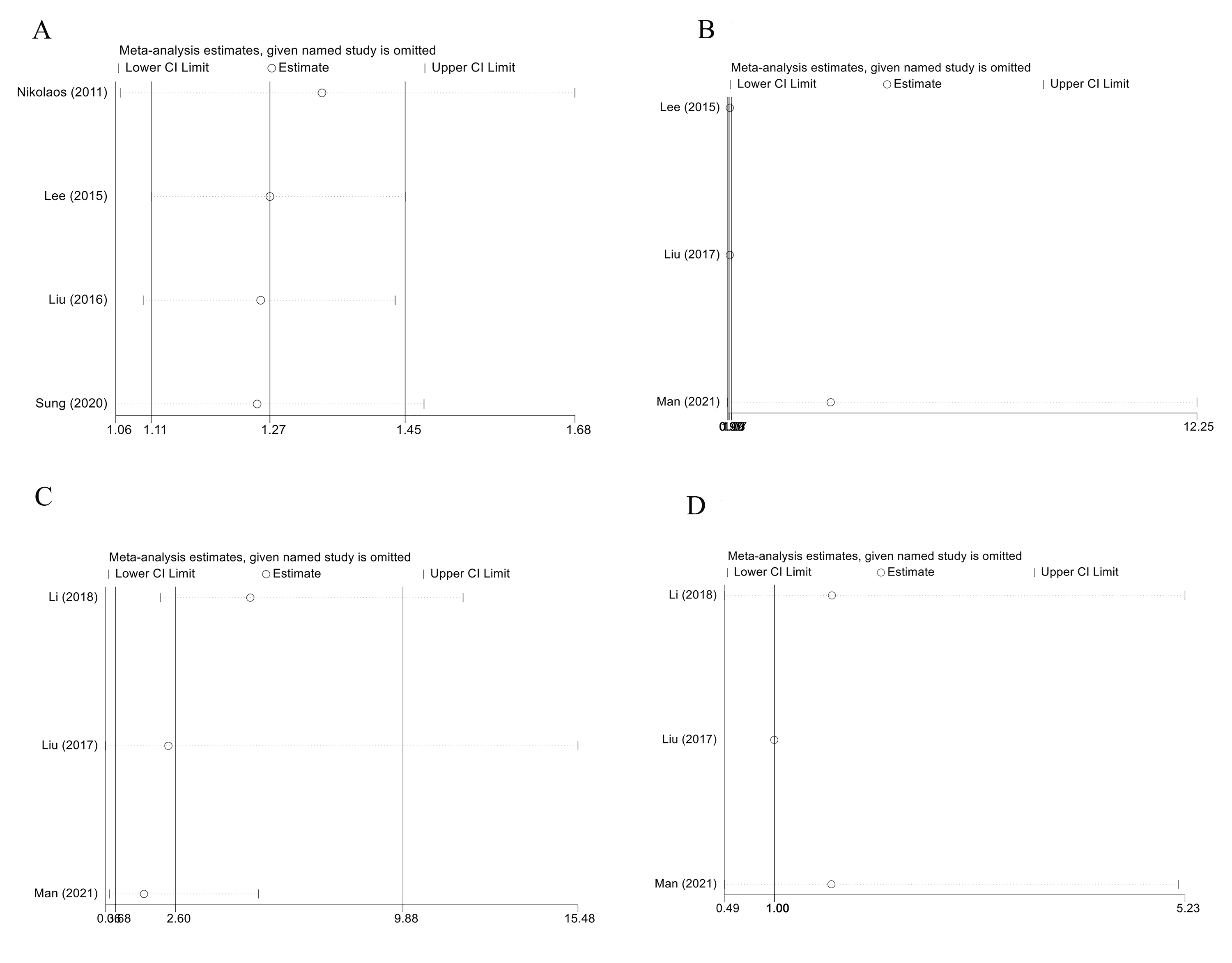

Supplement: Supplementary Figure S1 — Results of sensitivity analysis in OS based on SUVmax (A), PFS based on SUVmax (B), MTV (C), TLG (D). [file Image_1.tif]
